# Development of a feasible and portable electronic flag for near-real-time identification of renal replacement therapy in the Veterans Health Administration

**DOI:** 10.1017/ash.2025.10166

**Published:** 2025-10-23

**Authors:** Samuel W. Golenbock, Dipandita Basnet, Hillary J. Mull, Rebecca Lamkin, Kimberly Harvey, Marlena Shin, Judith M. Strymish, Sarah Leatherman, Ryan Ferguson, Westyn Branch-Elliman

**Affiliations:** 1 VHA Boston Healthcare System, Center for Health Optimization and Implementation Research (CHOIR), Boston, MA, USA; 2 Boston University School of Public Health, Boston, MA, USA; 3 Department of Surgery, Boston University Chobanian & Avedisian School of Medicine, Boston, MA, USA; 4 Department of Medicine, Section of Infectious Diseases, Greater Los Angeles VA Healthcare System, Los Angeles, CA, USA; 5 Department of Medicine, UCLA David Geffen School of Medicine, Los Angeles, CA, USA; 6 Center for the Study of Healthcare Innovation, Implementation, and Policy (CSHIIP), Greater Los Angeles VA Healthcare System, Los Angeles, CA, USA; 7 VA Cooperative Studies Program, Boston, MA, USA; 8 Boston University Chobanian and Avedisian School of Medicine, Boston, MA, USA

## Abstract

**Background:**

Chronic kidney disease (CKD) is prevalent among US Veterans. Identifying patients undergoing dialysis in real-time is crucial for implementing patient safety measures, including stewardship interventions, such as medication dosing adjustments. Limited feasible and accurate tools exist for near-real-time identification. This study aimed to develop a renal replacement therapy (RRT) flag using structured data in the Veterans Health Administration (VHA) electronic health record (EHR).

**Methods:**

Data from Veterans who underwent cardiovascular implantable electronic device (CIED) procedures (9/2015–12/2019) were linked to US Renal Data Systems (USRDS) data. Potential identifiers included outpatient hemodialysis procedure records, community care hemodialysis consults, ICD-10 diagnoses, and serum creatinine (SCr) >4 mg/dL. USRDS served as the comparison standard, and sensitivity, specificity, positive predictive value (PPV), and negative predictive value (NPV) were calculated. Logistic regression determined the area under the curve (AUC).

**Results:**

Among 37,706 CIED procedures on 34,994 Veterans, 967 patients (2.6%) were identified by USRDS as ever receiving RRT (hemodialysis and peritoneal dialysis or transplant), with 520 (1.4%) actively receiving RRT at the time of CIED. The RRT flag, combining ≥4 outpatient procedures in the prior 30 days, ≥1 consult in the prior year, and/or SCr >4 mg/dL, achieved an AUC of 0.976 (95% CI: 0.97–0.98), with high sensitivity (0.96; 95% CI: 0.94–0.97) and specificity (0.99; 95% CI: 0.99–1.00). The PPV was 0.70 (95% CI: 0.67–0.74). Performance was slightly lower when consults were replaced with ICD codes.

**Conclusions:**

We developed an accurate electronic flag using structured data to identify active RRT within VHA among Veterans undergoing invasive procedures, supporting patient safety and care adjustments. This flag addresses a crucial patient safety gap and supports expansion of stewardship efforts.

## Background

Chronic kidney disease (CKD) and renal replacement therapy (RRT) are common among the US Veteran population.^1^ Identification of patients receiving RRT is an important patient safety consideration, as these patients have specialized care needs, such as medication adjustments and dialysis access care needs. For the purposes of antimicrobial stewardship and infection surveillance activities, identification of patients receiving dialysis is important for supporting appropriate antibiotic dosing and as part of the tools to identify harms associated with antimicrobial use, such as acute kidney injury (AKI).^2–6^ Notably, multiple members of the clinical care team (eg, pharmacists, nurses) provide specific specialized care to patients receiving dialysis and have a need to know about this information to act upon it, and yet systems for identifying these patients for all provider types are not systematically available.

Due to availability of dialysis centers in the community and the mechanisms of federal reimbursement for the services, most Veterans who undergo RRT receive their CKD care outside of the Veterans Health Administration (VHA) healthcare system.^7^ Because this care is provided outside of the VHA ecosystem, information about active RRT use is not easily available to providers with access only to the VHA’s electronic medical record, nor can coded data be obtained in the VHA Corporate Data Warehouse (CDW), the VA’s central data repository.^8^ Although the presence of stage V kidney disease may be coded in diagnostic data following discharge, there are limited tools available to identify patients *actively* receiving RRT when they are admitted to acute care facilities or present for surgical, emergency, or urgent care services.^9^ Similar challenges are likely to exist in other health systems that do not provide comprehensive RRT services and therefore may lack these records. Thus, the aim of this study was to develop a simple RRT flagging tool based on structured data elements that could be used to support real-time stewardship and antimicrobial harm surveillance activities within the VHA.

## Methods

### Overview

This study to develop and test an RRT flagging tool was part of a larger multicenter prospective implementation study to leverage informatics tools to reduce guideline-discordant antimicrobial use in the electrophysiology laboratory.^10,11^ Specific elements of the informatics tools included audit-and-feedback about guideline concordance, cardiovascular implantable electronic device (CIED) infection rates, and antimicrobial harms. Harms included AKI and dialysis events.^11^

With the existing national cohort of Veterans undergoing CIED procedures (implantations and revisions of permanent pacemakers, implantable cardioverter defibrillators [ICDs], biventricular pacemaker ICDs) from September 1, 2015, to December 31, 2019,^12^ we linked patient identifiers to US Renal Data Systems (USRDS) RRT utilization data.^13^ The USRDS maintains detailed, validated data about RRT utilization (hemodialysis, peritoneal dialysis, unclassified) and outcomes among patients with end-stage renal disease (ESRD), including transplantation status.^13^ Although these data are highly accurate and curated, availability is substantially delayed, lagging up to 2 years before information is available for utilization.^14^ The 2-year lag prohibits the use of USRDS data for the purposes of adverse event surveillance and quality improvement initiatives.^13^ Potential flags were created from structured data elements extracted from the VHA CDW. Criterion validity was tested using the USRDS data set as the gold standard.

Patient characteristics and procedure details were accessed from the CDW. Data were analyzed between September 1, 2022, and July 1, 2023. The VHA Boston Institutional Review Board approved this study prior to data abstraction and analysis.

### RRT electronic flag development

Structured data elements in the VHA CDW potentially associated with receipt of RRT in our sample of Veterans undergoing CIED procedures included (1) outpatient dialysis procedure records, (2) VHA dialysis consults to community care encounters, (3) inpatient and outpatient ICD-10 diagnosis codes, and (4) preoperative serum creatinine (SCr) levels from VHA laboratory test results.

We first identified Current Procedural Terminology (CPT) codes for receipt of outpatient dialysis treatment within the VHA (see Supplemental Table A for the full list of codes). Based on consensus from VHA nephrology providers, patients with 4 or more treatment days in the 30-day period prior to their CIED procedure were classified as current RRT patients (internal communication).

Next, we reviewed prior RRT specialty consults to identify patients who may receive RRT primarily in non-VHA community care settings. This variable included multiple lookback periods prior to CIED procedure date: 90, 180, and 365 days. To evaluate portability of the tool (eg, utility in non-VHA settings), we also examined a version of the flag using ICD-10 diagnosis codes for severe and/or moderate renal failure as a substitute for VHA consults. Lastly, mean SCr levels from at least 2 laboratory tests during the 6 months prior to procedure were dichotomized at different threshold values: 3.0, 4.0, and 5.0 mg/dL.

Combinations of structured data elements for the RRT flag were used to create an aggregate measurement tool, where the presence of at least 1 of the 3 elements was considered sufficient to assign active dialysis status. Data sources accessed included administrative files (procedure codes, patient characteristics) and structured tables (consult requests, laboratory test results). Data were extracted from the VHA electronic health records (EHR) data (CDW) using SQL Server Management Studio 18 (Microsoft Corp) and analyzed using SAS 9.4.

### USRDS data set

Data on ESRD patients were retrieved from the USRDS 2021 core data set.^13,14^ The USRDS core data set contains patient demographic information, payer and treatment history, and transplant data on veterans and non-veterans who have initiated treatment for CKD and/or ESRD.^9^ We used the USRDS Patient Profile and Condensed Treatment History files to identify patients who were on an active RRT at the time of their cardiac device procedure. Active RRT modalities included “Center hemodialysis (HD),” “Center Self HD,” “Home HD,” “Continuous Ambulatory Peritoneal hemodialysis (CAPD),” “Continuous Cycling Peritoneal hemodialysis (CCPD),” “Other PD,” and “Uncertain hemodialysis.” Patients who were absent from the USRDS database, or whose device placements occurred during a period of “Discontinued hemodialysis,” “Functioning transplant,” “Lost to follow-up,” or “Recovered function” were not classified as true hemodialysis cases.

### Statistical analysis

Potential flags were iteratively tested against an indicator for USRDS-verified active RRT modality at the time of CIED procedure (ie, true RRT case). Measures of criterion validity (sensitivity, specificity, positive predictive value [PPV], and negative predictive value [NPV]) were assessed for each combination of structured data elements against the USRDS results. Test flags were evaluated with and without VA-specific data elements (eg, with and without community consults and ICD codes), and measures of criterion validity were compared to assess the potential for the tool to be useful for implementation in non-VHA settings. Logistic regression was used to calculate the predicted probability of RRT for each validation measure, and areas under the curve (AUC; equivalent to c-statistic for binary models) were compared between different versions of the RRT flag. A final version was selected for use in both VHA and non-VHA settings after evaluating flags based on criterion validity characteristics, AUC, and the precision of the test flag rate relative to the true USRDS rate. We also assessed whether the flag was appropriately classifying patients who had undergone prior renal transplantation and were no longer receiving RRT.

To assess algorithmic equity, the model was also evaluated stratified by race and sex, and AUCs were compared across different demographic groups.

## Results

During the period from September 2015 to December 2019, 37,706 CIED procedures were performed on 34,994 unique Veterans. In total, 976 patients ever receiving RRT were identified in the USRDS data set (2.5%). Among the 37,706 CIED procedures included in the study, 520 (1.4%) had a USRDS record of an active RRT treatment modality at the time of the procedure; among these cases, 94% were receiving hemodialysis, 5% peritoneal dialysis, and 1% unclassified. There were 103 patients who had a prior renal transplant.

Patient demographic and other procedure-related characteristics are presented in Table 1. The vast majority of the patients included in the cohort were male. Patients receiving RRT were 69.8 years (standard deviation [*SD*] = 9.4), and patients not receiving RRT were 72.3 years (*SD* = 9.4). Patients receiving RRT were disproportionately black (38.8%) versus those not receiving RRT (15.5%) and were more likely to have received an ICD-10 diagnosis of severe renal failure (76.5%) versus patients not receiving RRT (1.9%). Among patients who were receiving RRT, 31.% received RRT care in VHA outpatient settings, and dialysis consults within the past year were common (76.5%). Most patients receiving RRT had an SCr >4.0.

**Table 1.** VHA patient characteristics at time of procedure by dialysis status, October 2015–December 2019

	Hemodialysis status^†^	
	No (n=37,186)	Yes (n=520)
**Patient Characteristics at Time of Procedure**		
Age (mean, SD)	72.3 (10.3)	69.8 (9.4)
Age group, n (%)		
• <50	799 (2.1)	7 (1.3)
• 50–64	6,237 (16.8)	119 (22.9)
• 65–74	16,357 (44.0)	260 (50.0)
• 75–84	8,919 (24.0)	101 (19.4)
• ≥85	4,874 (13.1)	33 (6.3)
Female, n (%)	797 (2.1)	8 (1.5)
Race, n (%)		
• White	28,710 (77.2)	262 (50.4)
• Black	5,782 (15.5)	202 (38.8)
• Asian	483 (1.3)	13 (2.5)
• Native or Pacific Islander	286 (0.8)	6 (1.2)
• Unknown/declined	1,925 (5.2)	37 (7.1)
Hispanic or Latino ethnicity, n (%)	2,095 (5.6)	43 (8.3)
Married, n (%)	19,016 (51.1)	261 (50.2)
Service-connected disability, n (%)	20,849 (56.1)	294 (56.5)
**Renal Replacement Therapy Characteristics at Time of Procedure**		
Comorbid diagnosis of renal failure, n (%)		
• ICD-10 diagnosis of renal failure (moderate)	3,956 (10.6)	6 (1.2)
• ICD-10 diagnosis of renal failure (severe)	692 (1.9)	398 (76.5)
• ICD-10 diagnosis of renal failure (moderate or severe)	4,648 (12.5)	404 (77.7)
Dialysis outpatient procedures (4 or more, past 30 days), n (%)	34 (0.1)	164 (31.5)
Dialysis consult (past year), n (%)	96 (0.3)	398 (76.5)
Mean serum creatinine >4.0 mg/dL, n (%)	128 (0.3)	412 (79.2)

^†^Patients who were undergoing renal replacement therapy at the time of their CIED procedure, according to the US Renal Data System (USRDS) database.

Potential structured data elements for the RRT flag (CPT codes, consults for dialysis, ICD-10 diagnoses, and creatinine levels) were all highly associated with ongoing receipt of dialysis, with AUCs for each of these individual components ranging from 0.657 to 0.918. Criterion validity measures of the different dialysis flags that were tested are presented in Table 2. The optimal version of the RRT flag (AUC = 0.976, 95% confidence interval [CI]: 0.97–0.98) included ≥4 outpatient procedures in the prior 30 days, 1 or more dialysis consult(s) in the prior year, and/or an average preprocedure SCr level >4.0 mg/dL. Among the 37,706 device procedures included in the study, 707 (1.9%) were flagged by the final combination measure, indicating that the variable components slightly overestimated the true incidence of RRT (1.4%) in this cohort. The sensitivity of the optimal algorithm was 0.96 (95% CI: 0.94–0.97), specificity was 0.99 (95% CI: 0.99–1.00), PPV was 0.70 (95% CI: 0.67–0.74), and NPV was 0.99 (95% CI: 0.99–1.00).

**Table 2.** Criterion validity measures for dialysis component and test flags

Flag description	Test flag rate	Sensitivity	Specificity	Predictive value (+)	Predictive value (−)	AUC
***Components***						
Dialysis outpatient procedures (4 or more, past 30 days)	0.5%	31.5%	99.9%	82.8%	99.1%	0.657
Dialysis consult (past 90 days)	0.7%	38.1%	99.9%	80.5%	99.1%	0.690
Dialysis consult (past 180 days)	1.0%	56.7%	99.8%	81.7%	99.4%	0.783
Dialysis consult (past 365 days)	1.3%	76.5%	99.7%	80.6%	99.7%	0.881
Mean serum creatinine >3.0 mg/dL	2.4%	84.8%	98.8%	49.1%	99.8%	0.918
Mean serum creatinine >4.0 mg/dL	1.4%	79.2%	99.7%	76.3%	99.7%	0.894
Mean serum creatinine >5.0 mg/dL	1.0%	64.2%	99.9%	86.8%	99.5%	0.820
ICD-10 Diagnosis of renal failure (moderate)	13.4%	77.7%	87.5%	8.0%	99.6%	0.826
ICD-10 Diagnosis of renal failure (severe)	2.9%	76.5%	98.1%	36.5%	99.7%	0.873
***Test Flags (VHA Data)***						
CPT counts + 90-day Consult + SCr 3.0 mg/dL	2.5%	90.8%	98.7%	49.5%	99.9%	0.947
**CPT counts + 90-day Consult + SCr 4.0 mg/dL**	**1.7%**	**88.7%**	**99.5%**	**73.2%**	**99.8%**	**0.941**
CPT counts + 90-day Consult + SCr 5.0 mg/dL	1.4%	81.9%	99.7%	80.1%	99.7%	0.908
CPT counts + 180-day Consult + SCr 3.0 mg/dL	2.6%	92.7%	98.7%	49.6%	99.9%	0.957
CPT counts + 180-day Consult + SCr 4.0 mg/dL	1.7%	91.0%	99.5%	71.9%	99.9%	0.952
CPT counts + 180-day Consult + SCr 5.0 mg/dL	1.5%	86.3%	99.7%	78.5%	99.8%	0.930
CPT counts + 365-day Consult + SCr 3.0 mg/dL	2.7%	96.3%	98.6%	49.6%	99.9%	0.975
CPT counts + 365-day Consult + SCr 4.0 mg/dL	1.9%	95.8%	99.4%	70.4%	99.9%	0.976
CPT counts + 365-day Consult + SCr 5.0 mg/dL	1.7%	94.4%	99.6%	77.0%	99.9%	0.970
***Test Flags (EHR Data)***						
CPT counts + SCr 3.0 mg/dL	2.4%	87.1%	98.8%	49.5%	99.8%	0.929
CPT counts + SCr 4.0 mg/dL	1.5%	83.3%	99.6%	74.9%	99.8%	0.914
CPT counts + SCr 5.0 mg/dL	1.2%	73.3%	99.8%	83.7%	99.6%	0.865
CPT counts + SCr 3.0 mg/dL + ICD10 renal failure (moderate)	14.1%	94.2%	87.0%	9.2%	99.9%	0.906
**CPT counts + SCr 4.0 mg/dL + ICD10 renal failure (moderate)**	**13.8%**	**93.5%**	**87.4%**	**9.4%**	**99.9%**	**0.904**
CPT counts + SCr 5.0 mg/dL + ICD10 renal failure (moderate)	13.7%	91.7%	87.4%	9.3%	99.9%	0.896
CPT counts + SCr 3.0 mg/dL + ICD10 renal failure (severe)	3.8%	93.7%	97.5%	34.0%	99.9%	0.956
CPT counts + SCr 4.0 mg/dL + ICD10 renal failure (severe)	3.3%	92.9%	98.0%	39.1%	99.9%	0.954
CPT counts + SCr 5.0 mg/dL + ICD10 renal failure (severe)	3.2%	90.8%	98.1%	39.6%	99.9%	0.944

Selected model indicated in bold. ICD = International Classification of Diseases; CPT = Current Procedural Terminology; SCr = serum creatinine; EHR = electronic health record.

The optimal RRT flag for non-VHA settings included ≥4 outpatient procedures in the prior 30 days and/or an average preoperative SCr level >4.0 mg/dL (AUC = 0.914, 95% CI: 0.91–0.92). Among the total population, 578 (1.5%) were flagged by the non-VHA flag. Sensitivity was 0.83 (95% CI: 0.80–0.87), specificity was 1.00 (95% CI: 0.99–1.00), PPV was 0.75 (95% CI: 0.71–0.79), and NPV was 0.99 (95% CI: 0.99–1.00). The non-VHA test flags combining with ICD-10 diagnoses of renal failure in place of VHA consults provided increased sensitivity (90.8%–94.2%), but with a marked corresponding reduction in PPV (9.2%–39.6%).

The final selected flags also performed well after stratifying by sex (Supplementary Tables 2 and 3) and race and ethnicity groups (Supplementary Table 4). The final flagging tool also correctly identified 96.1% of active transplant patients as not receiving RRT (100/103).

## Discussion

End-stage kidney disease is a common clinical problem with significant impacts on all aspects of clinical care delivery; patients receiving RRT often require medication adjustments, referral for kidney transplant evaluation, specialized line care, and other interventions. These clinical care activities are provided by a variety of different types of care providers, including physicians, nurses, and pharmacists, among others. However, despite the frequency of RRT, there are limited informatics tools for identifying patients receiving these services so that patient safety interventions such as medication safety reviews can be deployed. We aimed to close that gap by developing an informatics-based solution that leverages structured data available in the EHR in near-real time to flag these patients. The AUC of the optimal measurement tool was .976, with very high PPV (70%), indicating outstanding predictive value for a variety of real-time or near-real-time uses.

This tool for identifying RRT was developed as part of a larger study to support expansion of infection prevention and antimicrobial stewardship services to areas with limited access to these resources.^11^ We hypothesized that antimicrobial use in these settings is driven in part by a perception that outpatient antimicrobial prescriptions are not associated with significant harm but provide benefit in terms of preventing infection.^8,11^ In this context, this tool was developed to support identification of antibiotic-associated acute kidney injuries, a relatively common complication of postprocedure and postoperative antimicrobial use,^4^ as part of a surveillance strategy presenting both benefits and harms of antimicrobial prescriptions. The tool has additional benefits of potential applications for real-time medication adjustments among patients with AKI who may need frequent changes to their antimicrobial dosing.

Management of RRT often occurs in outpatient and community settings, and thus, identifying cases can be a challenge due to missing and incomplete medical records data. Because of the mechanisms of care delivery, this information can be difficult to obtain in near real-time for surveillance. Although in theory variables such as consult receipt indicate an awareness that a patient is receiving RRT, in this cohort, more than 20% of Veterans actively receiving RRT services would have been missed if this variable had been deployed as a stand-alone approach. Additionally, consult requests are not readily available or accessible to all members of the healthcare team and therefore would not on their own solve the problem of real-time flagging for adjusting clinical care. These problems are particularly acute outside of traditional inpatient settings, where access to comprehensive surveillance tools is often quite limited but patient safety concerns remain.

Accurate identification of patients actively receiving RRT is critical, given the increased risk of adverse outcomes these patients face, particularly in postoperative settings. Expansion of the EHR and advancements in data availability create opportunities for informatics-based solutions to challenges with information silos and care coordination in both VHA and non-VHA settings.^15^ For example, the flag for measuring RRT cases could be operationalized beyond the VHA. Modern EHRs collect CPT and ICD-10 codes as well as laboratory results that perform well as components in our dialysis flag.^16^ Gold standard data from USRDS provides a useful resource to test the flag developed in this study and identify the weaknesses of both using 1 flag alone (eg, CPT codes) and using large research databases that are available years after data are collected. The results highlight the deficiencies of relying solely on VHA diagnostic codes, which often fail to capture the full scope of patients receiving dialysis and simultaneously identify too broad a selection of patients with renal dysfunction. This inadequacy can lead to significant gaps in patient safety and care quality, particularly in CKD-related postoperative adverse events. This combined flag represents a substantial improvement over the use of coding data alone, in both the VA-specific and non-VHA-specific flags.

The RRT flag developed for near-real-time case identification performed well using both VA-specific flags (eg, community care consults) and without the VA-specific variables and across different races and ethnicities, highlighting the portability of the electronic flag and algorithmic equity. Notably, the flag’s performance was somewhat lower in female patients, although power was limited to assess true accuracy, given the small number of female patients included. However, it is also possible that this was a true finding due to lower average SCr levels in female patients and true differences in kidney disease progression in males and females.^17–19^ Recently updated tools for estimating glomerular filtration rate (eGFR) include a creatinine adjustment for female sex,^20^ and additional work in other populations is needed to determine if similar adjustments are needed to improve case ascertainment of RRT use in this population. To ensure accurate classification in this group, replication in another patient population is prudent.

Our study is limited in several ways. First, the study was conducted within the closed VHA healthcare system, which may have more complete longitudinal follow-up data than other EHRs. Thus, it is possible that tools developed in the VHA will not perform as well when applied in different healthcare settings. However, our flag is based on variables that are nearly universally available in any EHR, supporting portability and feasibility of expansion and use outside of the VHA system. Second, the participants in this study reflect the VHA CIED population. Overall, the VHA population is heavily skewed toward male patients, and white and black patients are over-represented compared to other racial and ethnic groups. However, in stratified analyses, the algorithm performed well across different racial and ethnic groups. It is possible that the flag would not perform as well if applied outside the VHA, particularly to systems with higher proportions of female patients, and adjustments based on sex should be evaluated before implementing the tool in other populations. An additional limitation to the development using this cohort is that patients undergoing invasive procedures may have more complete data, including preoperative and preprocedural laboratory testing, than other populations. Performance will be lower in groups without laboratory data available. Third, we opted to apply relatively simple and straightforward variables likely to be available, such as SCr level, rather than more complex variables based on additional calculations, such as the eGFR. It is possible that our tool could be improved with additional complexity. Finally, the flag was developed using retrospective data, which are inherently more complete and comprehensive than prospective, near-real-time data sets. It is possible that the algorithm may perform less well in real-world settings. However, we attempted to mitigate against this problem in the design of the tool, which includes dialysis consults as a proxy for community-based ESRD treatment and is based on only structured data elements such as recent laboratory results that are less likely to be delayed than clinical notes, which are at the discretion of individuals to complete.

## Conclusions

We developed an electronic flag based on structured data elements for identifying active RRT cases within the national VHA healthcare system with outstanding predictive value for procedural settings. This simple structured tool can be used to support a variety of real-time patient safety efforts, including medication dosing adjustments and surveillance tools to support identification of antimicrobial harms as part of a larger stewardship program.

## Supporting information

10.1017/ash.2025.10166.sm001 Golenbock et al. supplementary material Golenbock et al. supplementary material

## Data Availability

The data that support the findings of this study are available from VHA Boston Healthcare System, but restrictions apply, and they are not publicly available. Data are, however, available from the authors upon reasonable request and with permission of the VHA Boston Healthcare System Data Security and Privacy office.

## Supplementary material

The supplementary material for this article can be found at http://doi.org/10.1017/ash.2025.10166.
