# Supplementary material for: Development of a feasible and portable electronic flag for near-real-time identification of renal replacement therapy in the Veterans Health Administration
Source: Antimicrob Steward Healthc Epidemiol. 2025 Oct 23;5(1):e279. doi: 10.1017/ash.2025.10166 (PMC12571676; doi:10.1017/ash.2025.10166)
Supplement: Golenbock et al. supplementary material [file S2732494X25101666sup001.docx]

| **Supplementary Table 1.** Current Procedural Terminology **(**CPT) codes used for identifying outpatient dialysis treatment. | | |
| --- | --- | --- |
| 90918 | 90953 | 90978 |
| 90919 | 90954 | 90979 |
| 90920 | 90955 | 90982 |
| 90921 | 90956 | 90983 |
| 90922 | 90957 | 90984 |
| 90923 | 90958 | 90985 |
| 90924 | 90959 | 90988 |
| 90925 | 90960 | 90989 |
| 90935 | 90961 | 90990 |
| 90937 | 90962 | 90991 |
| 90939 | 90963 | 90992 |
| 90940 | 90964 | 90993 |
| 90941 | 90965 | 90994 |
| 90942 | 90966 | 90995 |
| 90943 | 90967 | 90996 |
| 90944 | 90968 | 90997 |
| 90945 | 90969 | 90998 |
| 90947 | 90970 | 90999 |
| 90951 | 90976 |  |
| 90952 | 90977 |  |

| **Supplementary Table 2.** Criterion validity measures for flags based on sex-dependent serum creatinine (SCr) levels among VHA cardiac device patients (n=37,706), October 2015 - December 2019, with 95% confidence intervals. | | | | | | | | | | | |
| --- | --- | --- | --- | --- | --- | --- | --- | --- | --- | --- | --- |
|  |  |  |  |  |  |  |  |  |  |  |  |
| **Flag description** | **Flagged** | **Test flag rate** (95% CI) | | **Sensitivity** (95% CI) | | **Specificity** (95% CI) | | **Predictive  value (+)** (95% CI) | | **Predictive  value (-)** (95% CI) | |
| Mean serum creatinine > 3.0 mg/dL | 898 | 2.4% | (2.2, 2.5) | 84.8% | (81.7, 87.9) | 98.8% | (98.7, 98.9) | 49.1% | (45.8, 52.4) | 99.8% | (99.7, 99.8) |
| Mean serum creatinine > 4.0 mg/dL | 540 | 1.4% | (1.3, 1.6) | 79.2% | (75.7, 82.7) | 99.7% | (99.6, 99.7) | 76.3% | (72.7, 79.9) | 99.7% | (99.7, 99.8) |
| Mean serum creatinine, sex-dependent cut-off (3.0 or 4.0 mg/dL)^a^ | 584 | 1.5% | (1.4, 1.7) | 85.4% | (82.4, 88.4) | 99.6% | (99.6, 99.7) | 76.0% | (72.6, 79.5) | 99.8% | (99.8, 99.8) |
| CPT counts + 365-day Consult + Sex-dependent SCr (3.0 or 4.0 mg/dL)^a^ | 722 | 1.9% | (1.8, 2.1) | 96.5% | (95.0, 98.1) | 99.4% | (99.3, 99.5) | 69.5% | (66.2, 72.9) | 100.0% | (99.9, 100.0) |
| CPT counts + Sex-dependent SCr (3.0 or 4.0 mg/dL)^a^ | 1,308 | 3.5% | (3.3, 3.7) | 97.7% | (96.4, 99.0) | 97.8% | (97.7, 98.0) | 38.8% | (36.2, 41.5) | 100.0% | (100.0, 100.0) |
| a. Sex-dependent serum creatinine (SCr) cut-offs were dichotomized at 3.0mg/dL and 4.0mg/dL for female and male Veterans, respectively. | | | | | | | | | | | |

| **Supplementary Table 3.** Criterion validity measures for dialysis components and test flags among VHA cardiac device patients (2015-2019), stratified by sex, with 95% confidence intervals. | | | | | | | | | | | | | |
| --- | --- | --- | --- | --- | --- | --- | --- | --- | --- | --- | --- | --- | --- |
|  |  |  |  |  |  |  |  |  |  |  |  |  |  |
| **Flag description** | **Sub- Group** | **Flagged / Total** | | **Test flag rate**  (95% CI) | | **Sensitivity**  (95% CI) | | **Specificity**  (95% CI) | | **Predictive  value (+)**  (95% CI) | | **Predictive  value (-)**  (95% CI) | |
| Mean serum creatinine (SCr) > 3.0 mg/dL | Female | 15 | 807 | 1.9% | (0.9, 2.8) | 87.5% | (64.6, 100.0) | 99.0% | (98.3, 99.7) | 46.7% | (21.4, 71.9) | 99.9% | (99.6, 100.0) |
|  | Male | 883 | 36,897 | 2.4% | (2.2, 2.6) | 84.8% | (81.7, 87.9) | 98.8% | (98.7, 98.9) | 49.2% | (45.9, 52.5) | 99.8% | (99.7, 99.8) |
| Mean serum creatinine (SCr) > 4.0 mg/dL | Female | 6 | 807 | 0.7% | (0.2, 1.3) | 62.5% | (29.0, 96.1) | 99.9% | (99.6, 100.0) | 83.3% | (53.5, 100.0) | 99.6% | (99.2, 100.0) |
|  | Male | 534 | 36,897 | 1.4% | (1.3, 1.6) | 79.5% | (76.0, 83.0) | 99.7% | (99.6, 99.7) | 76.2% | (72.6, 79.8) | 99.7% | (99.7, 99.8) |
| CPT counts + SCr 3.0 mg/dL | Female | 15 | 807 | 1.9% | (0.9, 2.8) | 87.5% | (64.6, 100.0) | 99.0% | (98.3, 99.7) | 46.7% | (21.4, 71.9) | 99.9% | (99.6, 100.0) |
|  | Male | 901 | 36,897 | 2.4% | (2.3, 2.6) | 87.1% | (84.2, 90.0) | 98.7% | (98.6, 98.9) | 49.5% | (46.2, 52.8) | 99.8% | (99.8, 99.9) |
| CPT counts + SCr 4.0 mg/dL | Female | 6 | 807 | 0.7% | (0.2, 1.3) | 62.5% | (29.0, 96.1) | 99.9% | (99.6, 100.0) | 83.3% | (53.5, 100.0) | 99.6% | (99.2, 100.0) |
|  | Male | 572 | 36,897 | 1.6% | (1.4, 1.7) | 83.6% | (80.4, 86.8) | 99.6% | (99.5, 99.7) | 74.8% | (71.3, 78.4) | 99.8% | (99.7, 99.8) |
| CPT counts + 365-day Consult + SCr 3.0 mg/dL | Female | 15 | 807 | 1.9% | (0.9, 2.8) | 87.5% | (64.6, 100.0) | 99.0% | (98.3, 99.7) | 46.7% | (21.4, 71.9) | 99.9% | (99.6, 100.0) |
|  | Male | 996 | 36,897 | 2.7% | (2.5, 2.9) | 96.5% | (94.9, 98.1) | 98.6% | (98.5, 98.7) | 49.6% | (46.5, 52.7) | 99.9% | (99.9, 100.0) |
| CPT counts + 365-day Consult + SCr 4.0 mg/dL | Female | 7 | 807 | 0.9% | (0.2, 1.5) | 75.0% | (45.0, 100.0) | 99.9% | (99.6, 100.0) | 85.7% | (59.8, 100.0) | 99.8% | (99.4, 100.0) |
|  | Male | 700 | 36,897 | 1.9% | (1.8, 2.0) | 96.1% | (94.4, 97.8) | 99.4% | (99.4, 99.5) | 70.3% | (66.9, 73.7) | 99.9% | (99.9, 100.0) |

| **Supplementary Table 4.** Criterion validity measures for final dialysis test flags among VHA cardiac device patients (2015-2019), stratified by race and ethnicity, with 95% confidence intervals. | | | | | | | | | | | | | |
| --- | --- | --- | --- | --- | --- | --- | --- | --- | --- | --- | --- | --- | --- |
|  |  |  |  |  |  |  |  |  |  |  |  |  |  |
| **Flag description** | **Sub-Group** | **Flagged / Total** | | **Test flag rate** (95% CI) | | **Sensitivity** (95% CI) | | **Specificity** (95% CI) | | **Predictive  value (+)** (95% CI) | | **Predictive  value (-)** (95% CI) | |
| CPT counts + 365-day Consult + SCr 4.0 mg/dL | White | 365 | 28,959 | 1.3% | (1.1, 1.4) | 96.2% | (93.9, 98.5) | 99.6% | (99.5, 99.7) | 69.0% | (64.3, 73.8) | 100.0% | (99.9, 100.0) |
|  | Black | 272 | 5,980 | 4.5% | (4.0, 5.1) | 95.5% | (92.7, 98.4) | 98.6% | (98.3, 98.9) | 71.0% | (65.6, 76.4) | 99.8% | (99.7, 100.0) |
|  | Hispanic/ Latino | 58 | 2,139 | 2.7% | (2.0, 3.4) | 97.7% | (93.2, 100.0) | 99.2% | (98.9, 99.6) | 72.4% | (60.9, 83.9) | 100.0% | (99.9, 100.0) |
|  | Other race/ ethnicity | 17 | 709 | 2.4% | (1.3, 3.5) | 100.0% | (100.0, 100.0) | 99.7% | (99.3, 100.0) | 88.2% | (72.9, 100.0) | 100.0% | (100.0, 100.0) |
|  | Unknown/ Declined | 49 | 1,980 | 2.5% | (1.8, 3.2) | 91.9% | (83.1, 100.0) | 99.2% | (98.8, 99.6) | 69.4% | (56.5, 82.3) | 99.8% | (99.7, 100.0) |
| CPT counts + SCr 4.0 mg/dL | White | 295 | 28,959 | 1.0% | (0.9, 1.1) | 83.2% | (78.7, 87.7) | 99.7% | (99.7, 99.8) | 73.9% | (68.9, 78.9) | 99.8% | (99.8, 99.9) |
|  | Black | 224 | 5,980 | 3.7% | (3.3, 4.2) | 83.2% | (78.0, 88.3) | 99.0% | (98.8, 99.3) | 75.0% | (69.3, 80.7) | 99.4% | (99.2, 99.6) |
|  | Hispanic/ Latino | 45 | 2,139 | 2.1% | (1.5, 2.7) | 83.7% | (72.7, 94.8) | 99.6% | (99.3, 99.9) | 80.0% | (68.3, 91.7) | 99.7% | (99.4, 99.9) |
|  | Other race/ ethnicity | 14 | 709 | 2.0% | (1.0, 3.0) | 86.7% | (69.5, 100.0) | 99.9% | (99.6, 100.0) | 92.9% | (79.4, 100.0) | 99.7% | (99.3, 100.0) |
|  | Unknown/ Declined | 43 | 1,980 | 2.2% | (1.5, 2.8) | 86.5% | (75.5, 97.5) | 99.4% | (99.1, 99.8) | 74.4% | (61.4, 87.5) | 99.7% | (99.5, 100.0) |
